# High Levels of Genomic Aberrations in Serous Ovarian Cancers Are Associated with Better Survival

**DOI:** 10.1371/journal.pone.0054356

**Published:** 2013-01-23

**Authors:** Lars O. Baumbusch, Åslaug Helland, Yun Wang, Knut Liestøl, Marci E. Schaner, Ruth Holm, Dariush Etemadmoghadam, Kathryn Alsop, Pat Brown, Australian Ovarian Cancer Study Group, Gillian Mitchell, Sian Fereday, Anna DeFazio, David D. L. Bowtell, Gunnar B. Kristensen, Ole Christian Lingjærde, Anne-Lise Børresen-Dale

**Affiliations:** 1 Department of Genetics, Institute for Cancer Research, Oslo University Hospital, The Norwegian Radium Hospital, Oslo, Norway; 2 Department of Oncology, Oslo University Hospital, The Norwegian Radium Hospital, Oslo, Norway; 3 Institute for Clinical Medicine, Faculty of Medicine, University of Oslo, Oslo, Norway; 4 Department of Gynecologic Oncology, Oslo University Hospital, The Norwegian Radium Hospital, Oslo, Norway; 5 Biomedical Informatics Group, Department of Informatics, University of Oslo, Oslo, Norway; 6 Centre for Cancer Biomedicine, University of Oslo, Oslo, Norway; 7 School of Medicine, Stanford University, Palo Alto, California, United States of America; 8 Department of Pathology, Oslo University Hospital The Norwegian Radium Hospital, Oslo, Norway; 9 Peter MacCallum Cancer Centre, Melbourne, Victoria, Australia; 10 Sir Peter MacCallum Department of Oncology, University of Melbourne, Melbourne, Victoria, Australia; 11 Department of Pathology, University of Melbourne, Melbourne, Victoria, Australia; 12 Westmead Institute for Cancer Research, University of Sydney at Westmead Millennium Institute and Department of Gynaecological Oncology, Westmead Hospital, Westmead, New South Wales, Australia; 13 Queensland Institute of Medical Research, Brisbane, Queensland, Australia; 14 Department of Biochemistry and Molecular Biology, University of Melbourne, Melbourne, Victoria, Australia; 15 Department of Pathology, University of Melbourne, Melbourne, Victoria, Australia; 16 Institute for Medical Informatics, Oslo University Hospital, The Norwegian Radium Hospital, Oslo, Norway; University of Texas Health Science Center at San Antonio, United States of America

## Abstract

Genomic instability and copy number alterations in cancer are generally associated with poor prognosis; however, recent studies have suggested that extreme levels of genomic aberrations may be beneficial for the survival outcome for patients with specific tumour types. We investigated the extent of genomic instability in predominantly high-grade serous ovarian cancers (SOC) using two independent datasets, generated in Norway (n = 74) and Australia (n = 70), respectively. Genomic instability was quantified by the Total Aberration Index (TAI), a measure of the abundance and genomic size of copy number changes in a tumour. In the Norwegian cohort, patients with TAI above the median revealed significantly prolonged overall survival (p<0.001) and progression-free survival (p<0.05). In the Australian cohort, patients with above median TAI showed prolonged overall survival (p<0.05) and moderately, but not significantly, prolonged progression-free survival. Results were confirmed by univariate and multivariate Cox regression analyses with TAI as a continuous variable. Our results provide further evidence supporting an association between high level of genomic instability and prolonged survival of high-grade SOC patients, possibly as disturbed genome integrity may lead to increased sensitivity to chemotherapeutic agents.

## Introduction

Serous ovarian cancers (SOC) are highly aggressive but often chemosensitive tumours, characterised by substantial morphological heterogeneity, frequent genomic aberrations, and genomic instability (see reviews by [1]–[3]). Most patients are diagnosed at an advanced stage of the disease [4], and almost half of all women (46%) diagnosed with SOC die within five years (http://seer.cancer.gov). Clinical and pathological classification methods, including tumour grade and the extent of surgical debulking, still fail to fully predict disease progression and patient outcome.

Microarray-based gene-expression profiling of tumours has been used to discriminate between patients with good or unfavourable prognosis and to categorize pathways for new treatment strategies in epithelial ovarian cancer [5]–[12]. Previous studies have identified genomic regions of frequent copy number change and mapped potential driver genes in high grade serous, clear cell, and mucinous ovarian tumours [13]–[16]. Further, amplified genes, including *RAB25* and *CCNE1*, have been associated with clinical parameters including histology, stage of the disease, outcome, or therapy response [17]–[22]. Although there has been some progress, prediction of clinical outcome for patients with SOC remains imprecise and challenging.

Genomic instability is a hallmark of malignant tumours, causing disturbed integrity of the genome, numerical alterations, and structural changes. For various cancer types greater genomic instability has been associated with poor prognosis, suggesting that genomic instability may confer growth advantage of cancer cells [23]–[25]. However, the effects of disordered genomic organization, including defects in the regulation of mitoses, chromosomal segregation, and spindle assembly, may also have an unfavourable effect on the overall viability and fitness of cancer cells [26], [27]. Consequently, there may be a critical level at which the disadvantageous effects of genomic instability on patient survival are outweighed by the detrimental effects on cancer cell viability. This hypothesis is supported by recent studies on survival in breast, ovarian, and other cancers, indicating a beneficial effect of extreme genomic instability [28], [29]. However, in most of these studies genomic instability has only been estimated indirectly on the basis of gene expression based signatures.

The capacity to repair genomic damage is crucial for cells to react on DNA damaging agents. Allelic imbalance or mutations in key checkpoint proteins result in impaired DNA repair and thus suggests increased sensitivity to DNA damaging chemotherapeutic drugs [30]. Thus, the extent of copy number variation may be an indicator of malignancy on one hand and sensitivity to therapy on the other. However, to measure directly the DNA repair capacity of cell lines or clinical specimens is difficult to perform, since the current genetic assays still lack high specificity [31].

In this study, we applied a numeric measure of genomic instability, which we termed the Total Aberration Index (TAI), to assess the level of genomic aberrations in SOC. Based on high-throughput DNA copy number data, we investigated the relationship between survival and the degree of genomic instability within two independent datasets of predominantly high-grade SOC patients.

## Materials and Methods

### Ethics statement

The study including patients of the Norwegian cohort was approved by the Regional Committees for Medical and Health Research Ethics (REC) board (Reference No: S-01127). Exception from written informed consent was given from the REC authorities based on patients being deceased and all materials used were remaining material after diagnosis. The study including patients of the Australian cohort was approved by the Human Research Ethics Committees at the Peter MacCallum Cancer Centre, Queensland Institute of Medical Research, University of Melbourne and all participating hospitals. Written informed consent was obtained from all participants in this study.

### Patient population and clinicopathological data

The Norwegian cohort, diagnosed and treated at the Department of Gynecological Oncology at the Oslo University Hospital The Norwegian Radiumhospital during the period May 1992 to February 2003, consisted of 74 patients diagnosed with SOC on routine pathology reports. All patients underwent primary surgery, followed by adjuvant platinum-based chemotherapy. A summary of the clinicopathological characteristics is shown in Table 1 and detailed information is provided in Table S1 (see also [32]). Progression-free survival (PFS) was defined as the time interval that elapsed between diagnosis and progression, based on the first confirmed sign of disease recurrence according to Gynecologic Cancer InterGroup (GCIG) definitions. Overall survival was defined as the time interval that elapsed between diagnosis and death of any cause [32]. Sensitivity to platinum-based chemotherapy was defined as no relapse within six months after the completion of the treatment.

**Table 1:** Clinicopathological characteristics of the Norwegian and Australian SOC patients.

		Norwegian cohort				Australian cohort			
		All	TAI<med.^1^	TAI>med.^1^	p*	All	TAI<med.^1^	TAI>med.^1^	p*
**Patients**	Total cases	74 (100%)	37 (50%)	37 (50%)		70 (100%)	35 (50%)	35 (50%)	
**Age**	Mean (SD)	60 (11)	60 (11)	60 (10)		57 (11)	55 (12)	58 (9)	
	Range	38–81	39–79	38–81		23–80	23–78	44–80	
**Age groups**	<45	7 (10%)	4 (11%)	3 (8%)	0.711	6 (9%)	5 (14%)	1 (3%)	0.226
	45–55	15 (20%)	6 (16%)	9 (24%)		25 (36%)	12 (34%)	13 (37%)	
	>55	52 (70%)	27 (73%)	25 (68%)		39 (56%)	18 (51%)	21 (60%)	
**Stage**	II	3 (4%)	1 (3%)	2 (5%)	0.958	0 (0%)	0 (0%)	0 (0%)	0.462
	III (B+C)	50 (68%)	26 (70%)	24 (65%)		62 (89%)	30 (86%)	32 (91%)	
	IV	21 (28%)	10 (27%)	11 (30%)		8 (11%)	5 (14%)	3 (9%)	
**Grade**	1	3 (4%)	2 (5%)	1 (3%)	0.186	4 (6%)	2 (6%)	2 (6%)	0.656
	2	21 (28%)	7(19%)	14 (38%)		24 (34%)	10 (29%)	14 (40%)	
	3	50 (68%)	28(76%)	22 (60%)		40 (57%)	22 (63%)	18 (51%)	
**Chemotherapy**	Sensitive	51 (69%)	21 (57%)	30 (81%)	**0.043**	39 (56%)	17 (49%)	22 (63%)	0.336
	Resistant	23 (31%)	16 (43%)	7 (19%)		31 (44%)	18 (51%)	13 (37%)	
**Progression**	Progression	69 (93%)	36 (97%)	33 (89%)	0.358	63 (90%)	3 (9%)	4 (11%)	1
	No progression	5 (7%)	1 (3%)	4 (11%)		7 (10%)	32 (91%)	31 (87%)	
**PFS (months)**	Median	16	15	18		15	12	19	
	(95% CI)	14–21	10–18	15–26		11–20	10–19	13–23	
**OS (months)**	Median	32	25	50		40	25	47	
	(95% CI)	25–47	17–31	34–67		28–54	19–57	35–60	

^1^Genomic instability was quantified as below (TAI<med.) or above (TAI>med.) median TAI. The median was 0.135 for the Norwegian cohort and 0.242 for the Australian cohort.

* Calculated p-values for age, stage, and grade from Mann-Whitney tests and for chemotherapy and progression from Fisher's exact tests.

**Abbreviations:** SOC, serous ovarian cancers; TAI, Total Aberration Index; PFS, progression-free survival; OS, overall survival; CI, confidence interval.

The second cohort, originally analysed in Australia [33], consisted of 70 patients diagnosed with SOC from 1988 to 2005, including 56 cases from Australia (from the Australian Ovarian Cancer Study (AOCS) and the Gynaecological Oncology Biobank at Westmead) and 14 cases from Japan. All patients received first-line platinum-based chemotherapy. A summary of the clinicopathological characteristics is shown in Table 1 and additional information is provided in Table S2 (further genetic information can be provided from AOCS Group on request). For this cohort, PFS was defined as the time interval between the date of diagnosis and the first confirmed sign of disease progression based on GCIG definitions. Overall survival was defined as the time interval between the date of histological diagnosis and the date of death from any cause [34]. Chemotherapy response was stratified based on progression-free interval; less than six months to disease progression was chosen as an end point to define resistant cases due to its clinical relevance in identifying platinum resistance [33].

### DNA extraction and copy number profiling

Haematoxylin and Eosin stained sections from frozen tissue were used to evaluate the percentage of tumour cells in tissue samples. The percentage of tumour cells in the samples in the Norwegian cohort ranged from 20% to 90% with a median of 70%. In the Norwegian cohort, genomic DNA was extracted from 10–15 serial frozen tissue sections (each 50 µm thick) using proteinase K digestion and phenol/chloroform in an ABI DNA extractor (Nucleic Acid Extractor 340A, applied Biosystems, Carlsbad, CA, USA) following standard protocols. Copy number profiles of all samples were obtained with the Stanford 42k cDNA aCGH platform (www.microarray.org/sfgf/jsp/home.jsp; for details see Materials and Methods S1). Data are stored in the GEO database with the accession number GSE35783.

Haematoxylin and Eosin stained sections from frozen tissue were also used to evaluate the percentage of tumour cells in tissue samples of the Australian cohort. Genomic DNA of samples was extracted from whole tumour tissue for samples with at least 80% neoplastic cells. For samples with less than 80% overall tumour cells needle dissection of serial tumour sections was done to enrich for epithelial fractions prior to DNA extraction. In the Australian cohort, DNA was extracted using DNeasy kit (Qiagen, Hilden, Germany) according to the manufacturer's protocol. Affymetrix 50 k *XbaI* single nucleotide polymorphism (SNP) (Affymetrix, Santa Clara, CA, USA) mapping arrays were applied to obtain copy number profiles (for details see Materials and Methods S1 and [33]). Data are stored in the GEO database with the accession number GSE13813.

### Segmentation and estimation of copy number data

To segment the copy number data the Piecewise Constant Fitting (PCF) algorithm [35]–[37] was applied to log2-transformed copy number values for each sample. For a given number of breakpoints, PCF identifies the least-squares optimal segmentation of the data. The number of breakpoints, and thus the bias-variance trade-off, is controlled by a penalty parameter 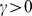 (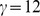 in this study). The least number of probes in a segment was set to 3. For each segment a corresponding (log2-transformed) segment average was obtained as the mean the log2-transformed copy number values for the probes in the segment.

### Assessing the genomic instability

The degree of genomic instability in a tumour was quantified by the total aberration level, using a similar method as described previously [38]. Let 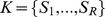 denote the segmentation obtained with PCF for a particular sample, where 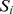 is the indices of the probes belonging to the i'th segment. Let 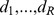 designate the segment length (in nucleotides) and 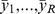 the corresponding segment averages. The Total Aberration Index (TAI) is then defined as
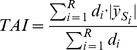



**Figure 1:**
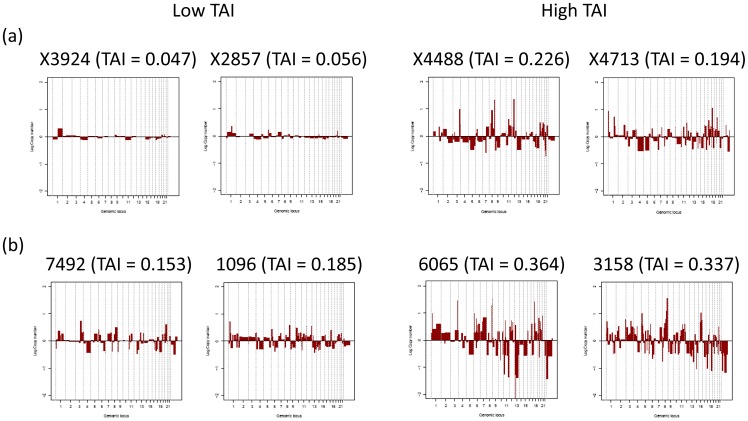
Examples of genomic profiles with low (left) and high (right) median Total Aberration Index (TAI). (a.) Examples from the Norwegian and (b.) from the Australian cohort. The log2-transformed copy numbers of the chromosomes 1 to 23 are illustrated. The median was 0.135 for the Norwegian and 0.242 for the Australian cohort.

Thus, TAI is basically a weighted sum of the segment averages and represents the absolute deviation from the normal copy number state, averaged over all genomic locations (for illustration see Figure S1 and for examples see Figure 1).

### Survival analysis

The Kaplan-Meier estimator and the log-rank test were used to obtain survival curves and to compare survival rates in patients with TAI below and above the median. To investigate the relationship between survival and TAI as a continuous variable, Cox proportional hazard models were fitted with TAI as the predictor. Analyses were performed separately on the Norwegian and Australian cohort.

All computations were performed using the statistical system R (v 2.12.2).

### Mutation testing

Comprehensive germ-line testing for the Australian cohort was completed in a certified diagnostic pathology laboratory using sequencing and multiplex ligation-dependent probe amplification [39].

## Results

### Frequency of aberrations

**Figure 2:**
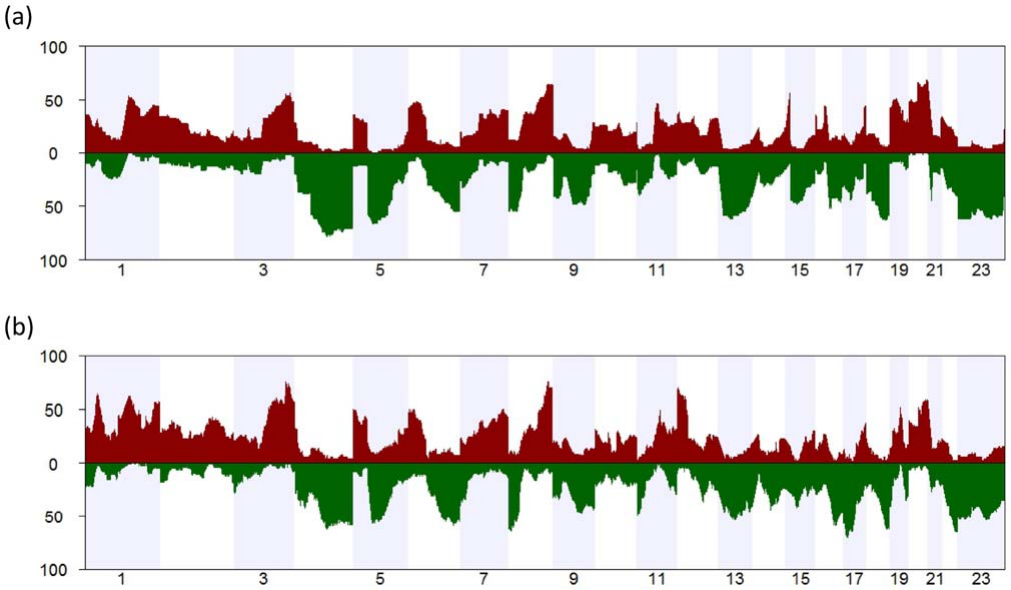
Frequency of copy number changes in serous ovarian carcinomas of two independent cohorts. The frequencies of copy number alterations in serous ovarian cancers of two independent cohorts from Norway and Australia are illustrated. Regions with copy number gains are marked in red and regions with copy number losses are marked in green, respectively. (a) The frequency of copy number changes of 74 serous ovarian tumours of the Norwegian cohort were determined using 42k cDNA arrays. Several high frequency peaks are visible, including gains at regions on chromosome arms 1q, 3q, 8q, and 20q, and losses on chromosome arms 4q, 5q, 6 p, 8 p, 13, 16q, 18q, and the whole of the X chromosome. (b) The frequency of aberrations of 70 ovarian tumour samples of the Australian cohort, as measured by 50 k SNP Affymetrix arrays. All high frequency peaks of the Norwegian cohort are also identified in the Australian cohort, although some additional peaks appear in the Australian data, e.g. gains in 1 p and losses on chromosome arms 17 p and 22q. The two data sets show high consistency in the aberration pattern, despite differences in populations and analysis platforms (see also Figure 3).

**Figure 3:**
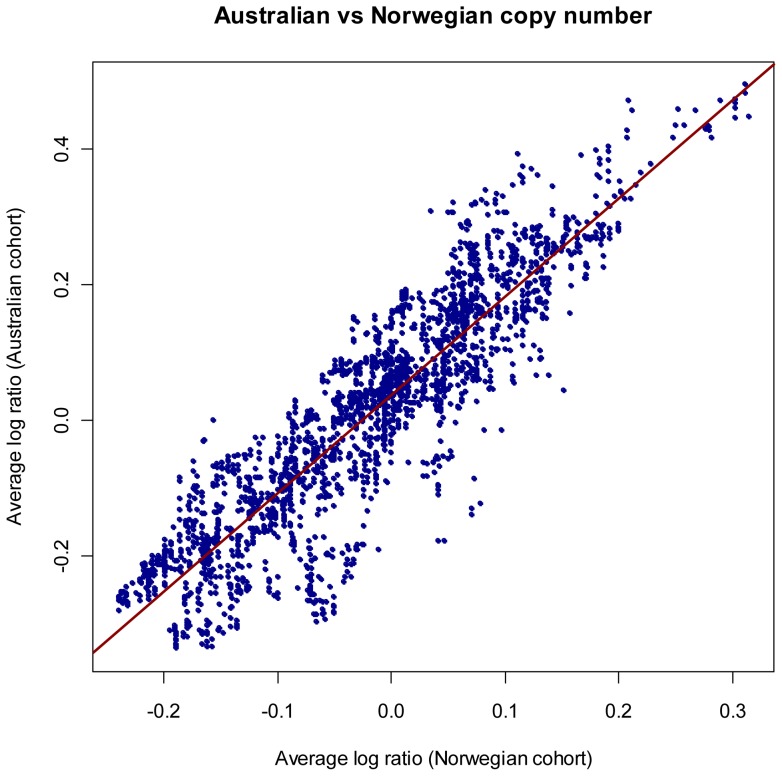
Comparison of estimated log copy numbers in the two cohorts. A total of 2923 genomic loci spaced 1Mb from each other were defined, and the average estimated log copy number was found at each loci and in each of the two study cohorts. The resulting set of 2923 pairs of averages is shown in the figure, suggesting considerable consistency between the two study cohorts.

The analysis of copy number data in serous ovarian cancers revealed that the aberrations in the Norwegian and Australian cohorts were broadly concordant (Figure 2 and Figure 3), with the most frequent gains occurring on chromosome arms 1q, 3q, 8q, and 20q, and the most frequent losses occurring on chromosome arms 4q, 5q, 6 p, 8 p, 13, 16q, 18q, and the whole of the X chromosome (Figure 2). In the Australian cohort, additional copy number gains were observed on 1 p and losses on 17 p and 22q (Figure 2b). The aberration patterns are also conform to those with high resolution arrays or sequencing data, reported elsewhere [7], [40].

### Survival analysis

**Figure 4:**
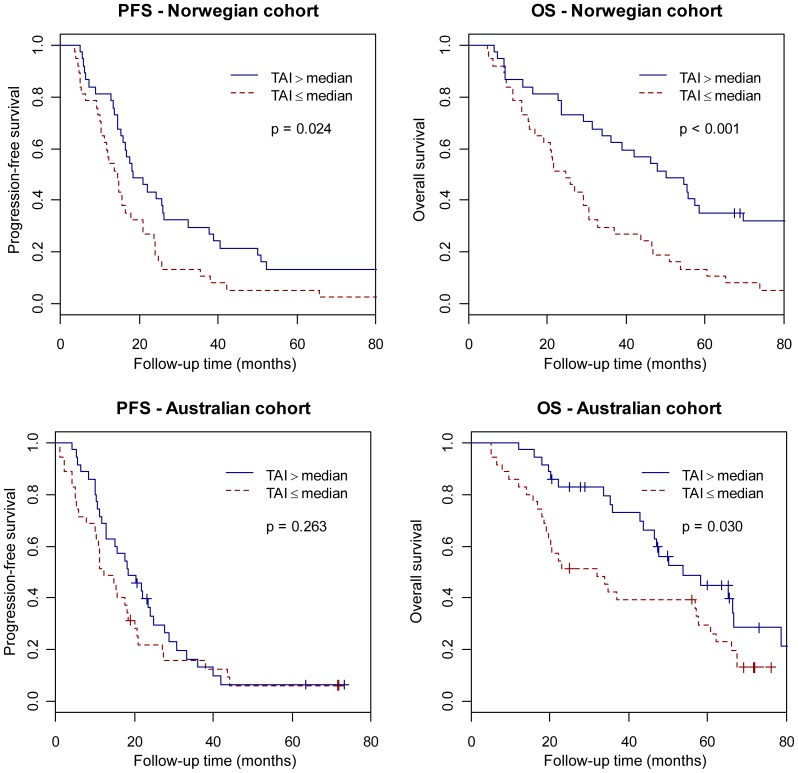
Survival analysis in relation to genomic instability. Kaplan-Meier survival curves illustrating progression-free survival (PFS) and overall survival (OS) time (in months) for serous ovarian cancers patients with Total Aberration Index (TAI) above and below the median in the Norwegian cohort (above) and the Australian cohort (below). Test results are based on log-rank tests. Note that high TAI implies a significant survival advantage, both with regard to progression-free survival and to overall survival in the Norwegian cohort, as well as for overall survival in the Australian cohort.

Figure 4 shows the analysis of progression-free survival and overall survival in patients with TAI greater or less than the median for the Norwegian cohort (median = 0.135) and Australian cohort (median = 0.242), respectively. In the Norwegian cohort, the group with TAI above the median had markedly increased progression-free survival (p = 0.024) and overall survival (p<0.001). In the Australian cohort, patients with TAI above the median had significantly increased overall survival (p = 0.030), while the progression-free survival was moderately, but non-significantly, prolonged. These results were confirmed by univariate Cox analysis, using TAI as a continuous variable (Table 2). In multivariate Cox analysis, which also included the variables age, stage, and grade; however, TAI was the only significant variable for both the Norwegian and Australian cohorts, suggesting that TAI is an independent predictor of clinical outcome (data not shown).

**Table 2:** Survival analysis of the Norwegian and Australian SOC patients.

	Progression-free survival		Overall survival	
Origin of data	Log-rank	Cox	Log-rank	Cox
**Norway**	P = 0.024	HR = 0.77 [0.62, 0.96]	p<0.001	HR = 0.70 [0.56, 0.88]
		p = 0.018		p = 0.001
**Australia**	P = 0.263	HR = 0.91 [0.70, 1.20]	p = 0.030	HR = 0.69 [0.51, 0.95]
		p = 0.498		p = 0.022

Log-rank: Log-rank tests comparing groups with above and below median TAI.

Cox: Cox proportional hazard regression with TAI as continuous variable.

HR: Hazard ratio with 95% confidence interval for an increase in TAI of 1SD.

### Genomic instability in relation to clinicopathological characteristics and mutation status

The clinicopathological characteristics age, stage, grade, chemotherapy response (stratified based on progression-free interval), and progression were analyzed in relation to differences in genomic instability (Table 1). Patients in the Norwegian cohort with TAI above the median showed a significantly (p = 0.043) higher sensitivity to chemotherapy compared to patients with TAI below the median. No other clinicopathological criteria were significantly different in the high or low TAI groups in the Norwegian cohort. In the Australian cohort, none of the investigated clinicopathological characteristics resulted in significant differences with regard to disparities in genomic instability.

Given that a germline mutation in either *BRCA1* or *BRCA2* in SOC patients is associated with favourable clinical outcome [39], [41], [42], and that these genes are involved in genome integrity, we tested whether TAI was a surrogate marker for carrier status. For the 35 patients in the Australian cohort with available *BRCA1* and *BRCA2* mutation status the germline carrier status was not significantly associated with the extent of genomic instability (using Fisher's exact test). Average TAI was 0.28 (SD = 0.06) for patients with germline mutation in *BRCA1* (n = 6), 0.27 (0.05) for patients with mutation in *BRCA2* (n = 2), 0.22 (0.03) for patients with unclassified variants of BRCA genes (n = 3), and 0.25 (0.05) for patients with wild type *BRCA* genes (n = 24).

## Discussion

We investigated the association between genomic instability and survival of predominantly high-grade SOC in two independent study cohorts from Norway and Australia and found that patients with high level of genomic instability, as measured by TAI, had a more favourable outcome. The results were confirmed by univariate and multivariate Cox analysis, were TAI was included as a continuous variable. The aberration patterns in the two cohorts, determined by two different gene-centred platforms, were highly concordant and consistent with those reported by others [14], [15], [20] and with data from The Cancer Genome Atlas project [7].

Only few publications have investigated the relationship between complex rearrangements and survival in ovarian cancer. A previous study, primarily focused on breast cancer but also considering ovarian tumours, provided some evidence that high levels of rearrangement in tumours may lead to better clinical outcome [28]. However, in that study genomic instability was based on the average expression of 70 genes that correlated with “total functional aneuploidy”.

The presented study is based on the analysis of high-resolution DNA copy number data and the application of a robust and easily interpretable measure of genomic instability (TAI). TAI assesses the deviation of the estimated copy number curve from the zero-line (Figure S1), and thus represents a numeric measure of the abundance and genomic size of copy number changes in a tumour. Low-grade ovarian tumours usually carry few genomic aberrations [43]; however, a small number of short aberrations in vital genes may be essential for initiating tumour development and progression. Such short aberrations have low impact on TAI making the index less suitable for studying initial steps in tumour development, but rather for quantifying the wide-spread genomic disorganization that may occur at a later stage of tumour progression. In the current work, we are considering advanced ovarian cancer with the aim of examining the importance of broad aberrations on survival and for this purpose TAI appears as a suitable way of obtaining numerical quantifications to be used in statistical analysis.

Genomic instability causes disturbed mitoses, segregation, and spindle assembly (see reviews by [44]–[46]). In ovarian cancer, as in other cancer types, genomic instability and copy number alterations have been associated with poor prognosis. However, recent publications have stated that high levels of genomic instability may be beneficial for the survival and prognosis of patients in some tumour types [28]–[30]. Furthermore, elevating the frequency of genomic instability has been proposed as a strategy to kill cancer cells [26].

It is thus possible that the initial growth advantage of cancer cells, based on the transforming effect of genomic instability, becomes a net disadvantage for the cancer cells, when the well-organized regulatory system is devastated. The capability for DNA repair may be reduced, leading to an increased sensitivity to DNA damaging agents, including chemotherapeutic drugs, such as cisplatin (see review by [47]). However, most patients are usually treated with adjuvant chemotherapy making it difficult to determine whether the observed association of genomic instability to patient survival is a result of intrinsically less fit cancer cells or the inability of the tumour cells to repair DNA damages caused by chemotherapeutic drugs. Thus, it is an interesting observation that in the Norwegian cohort the patients with a high degree of genomic instability showed a significantly better response to platinum-based chemotherapy.

SOC patients with germline mutations in *BRCA1* and *BRCA2* are more sensitive to chemotherapy and have improved survival [39], [41], [42]. In addition, an even higher fraction of ovarian cancer patients have somatic aberrations in the *BRCA* genes or the *BRCA*-pathway, characterising the phenotype called *BRCA*-ness [48]. A number of patients (n = 35) in the Australian cohort were analysed for germline *BRCA*-mutations. No significant difference in the TAI-index was observed between the *BRCA*-mutated samples and others, a finding that is consistent with the TCGA analysis of BRCA1/2 mutation and ploidy in a large series of SOC [39], [41], [42]. Germline status may only be represented in a fraction of the total homologous recombination dysfunction observed in the entire cohort, therefore making it difficult to associate homologous recombination deficiency with the extent of genomic aberration in tumours [7].

Precise delineation of the negative and positive effects of genomic instability on cancer cells is of potentially great importance for tumour classification, survival prediction, and individualized therapy [49]. However, the mechanisms of genomic instability transforming the initial advantageous effects on cancer cell survival into disadvantageous outcome are still unknown, likewise, how these mechanisms have potential influence on drug efficiency. Further studies, including other cancer types, are necessary to validate and refine the presented findings before the biological and clinical significance of genomic instability may be determined.

## Supporting Information

Materials and Methods S1 **Copy number profiling.**
(PDF)

Figure S1 **Model of the Total Aberration Index algorithm.**
(PDF)

Table S1 **Additional clinical data for the Norwegian cohort.**
(XLSX)

Table S2 **Additional clinical data for the Australian cohort.**
(XLSX)

Abstract S1 **Abstract in German.**
(PDF)

Abstract S2 **Abstract in Norwegian.**
(PDF)
