# Supplementary material for: High Levels of Genomic Aberrations in Serous Ovarian Cancers Are Associated with Better Survival
Source: PLoS One. 2013 Jan 23;8(1):e54356. doi: 10.1371/journal.pone.0054356 (PMC3553118; doi:10.1371/journal.pone.0054356)
Supplement: Materials and Methods S1 — Copy number profiling. (PDF) [file pone.0054356.s001.pdf]

Material and Methods S1: Copy number profiling.

Ninety samples were successfully analyzed by cDNA microarray-based CGH and of these 74 belonged to the group of serous carcinomas. Genomic DNA labelling and hybridizations were performed as described [1], with slight modifications. Briefly, two micrograms of tumour genomic DNA (test DNA) and normal female leukocyte DNA (reference DNA) were fluorescently labeled with Cy5 and Cy3, respectively, and hybridized overnight to a human cDNA microarray produced at the Stanford Functional Genomics Facility (www.microarray.org/sfgf/jsp/home.jsp) with 41,805 features representing more than 27,286 unique genes. After washing, the microarrays were scanned using a GenePix 4000 microarray scanner (Axon Instruments, Foster City, CA, USA); fluorescence ratios (tumour/reference), after background subtraction, were calculated using GenePix software. Only spots with fluorescence intensities more than 40% above background and for which this standard was met on at least 80% of the arrays were included in the further analyses. When the same known Unigene cluster was represented by multiple arrayed elements, log_2 mean fluorescence ratios were reported. Fluorescence ratios were normalized for each array by setting the average log_2 fluorescence ratio for all array elements equal to zero. Map positions for arrayed human cDNAs were assigned as described [3] and based on the "Golden Path" genome assembly (http://genome.ucsc.edu/; July, 2003 Freeze). Data are stored in the GEO database (accession number GSE35783).

The samples of the Australian cohort were analyzed on Affymetrix 50k *XbaI* single nucleotide polymorphism (SNP) (Affymetrix, Santa Clara, CA, USA) mapping arrays. Genomic DNA was digested with *XbaI* endonuclease, adapter ligated, and PCR amplified. PCR products were then purified, fragmented with *DNaseI*, biotin labeled, denatured, and hybridized to the array. Next, the arrays were washed on the Affymetrix fluidics station 450, streptavidin stained and scanned using a GeneChip® Scanner 3000. Data were extracted using Affymetrix GeneChip® Operating Software 1.4 (GCOS) and SNP genotypes were assigned using GeneChip® Genotyping Analysis Software 4.1 (GTYPE). Median call rate (the proportion of SNPs assigned a genotype) for tumor samples was 96.1% indicating high assay performance (for further information see [3]). Data are stored in the GEO database (accession number GSE13813).

References

1. Pollack JR, Perou CM, Alizadeh AA, Eisen MB, Pergamenschikov A et al. (1999) Genome-wide analysis of DNA copy-number changes using cDNA microarrays. Nat Genet 23: 41-46.

2. Pollack JR, Sorlie T, Perou CM, Rees CA, Jeffrey SS et al. (2002) Microarray analysis reveals a major direct role of DNA copy number alteration in the transcriptional program of human breast tumors. Proc Natl Acad Sci U S A 99: 12963-12968.

3. Etemadmoghadam D, deFazio A, Beroukhim R, Mermel C, George J et al. (2009) Integrated genome-wide DNA copy number and expression analysis identifies distinct mechanisms of primary chemoresistance in ovarian carcinomas. Clin Cancer Res 15: 1417-1427.
