# Supplementary material for: High Levels of Genomic Aberrations in Serous Ovarian Cancers Are Associated with Better Survival
Source: PLoS One. 2013 Jan 23;8(1):e54356. doi: 10.1371/journal.pone.0054356 (PMC3553118; doi:10.1371/journal.pone.0054356)
Supplement: Figure S1 — Model of the Total Aberration Index algorithm. (PDF) [file pone.0054356.s002.pdf]

Figure S1. Model of the Total Aberration Index algorithm.

The Total Aberration Index (TAI) reveals the absolute area under the curve defined by the Piecewise Constant Fit (PCF) obtained copy number profile:

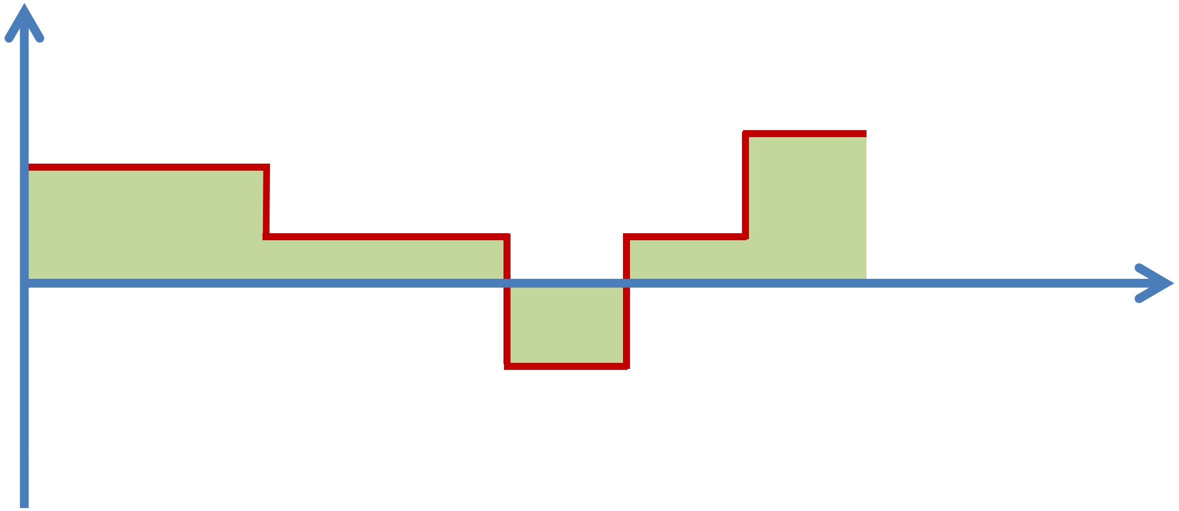

$$TAI = \frac{\sum_{i=1}^{R} d_i \cdot \left| \overline{y}_{S_i} \right|}{\sum_{i=1}^{R} d_i}$$

$\overline{y}_S$ = log2-transformed copy number value

$d_1, \ldots, d_R$ = genomic sizes of the segments

S_i = Indices of the probes belonging to the i'th segment
