# Supplementary material for: High Levels of Genomic Aberrations in Serous Ovarian Cancers Are Associated with Better Survival
Source: PLoS One. 2013 Jan 23;8(1):e54356. doi: 10.1371/journal.pone.0054356 (PMC3553118; doi:10.1371/journal.pone.0054356)
Supplement: Abstract S1 — Abstract in German. (PDF) [file pone.0054356.s005.pdf]

Abstract S1: Abstract in German.

In serösen Ovarialkarzinomen sind massive genomische Veränderungen mit besseren Überlebensprognosen assoziiert

Bei Krebserkrankungen sind genomische Instabilität und Veränderungen der DNA-Struktur normalerweise mit einer schlechteren Prognose verbunden. Neuere Studien deuten jedoch drauf hin, dass extrem ausgeprägte Veränderungen in einigen Tumorarten zu verbesserten Überlebensprognosen führen können. In dieser Studie haben wir das Ausmaß von Instabilität des Genoms in serösen Ovarialkarzinomen (SOK) in zwei unabhängigen Datensätzen, aus Norwegen (n=74) und Australien (n=70), untersucht. Dabei wurde die genomische Instabilität als Gesamtabweichungsindex (Total Aberration Index = TAI) quantifiziert, welcher die Häufigkeit und Umfang von Veränderungen der DNA-Struktur in Tumoren abschätzt. Im norwegischen Datensatz wiesen Patienten mit TAI Werten oberhalb des Medians eine signifikant verlängerte Totalüberlebensrate ($p<0.001$) und eine signifikant verlängerte rezidiv-freie Überlebensrate ($p<0.001$) auf. Im australischen Datensatz zeigten Patienten mit TAI Werten oberhalb des Medians ebenfalls eine signifikant verlängerte Totalüberlebensraten ($p<0.05$) und eine moderat verlängerte rezidiv-freie Überlebensrate, letztere war allerdings nicht signifikant. Die Ergebnisse wurden unter Verwendung des TAI als kontinuierlicher Variabler in univariaten und multivariaten Cox Regressionsanalysen bestätigt. Die Ergebnisse unserer Studie sind ein weitere Beleg dafür, dass sich ausgeprägte genomische Instabilität bei Pateinten mit SOK positiv auf die Überlebensprognosen auswirken können, vermutlich führt eine gestörte Genomintegrität zu erhöhter Sensibilität gegenüber chemotherapeutischen Substanzen.
