# Supplementary material for: High Levels of Genomic Aberrations in Serous Ovarian Cancers Are Associated with Better Survival
Source: PLoS One. 2013 Jan 23;8(1):e54356. doi: 10.1371/journal.pone.0054356 (PMC3553118; doi:10.1371/journal.pone.0054356)
Supplement: Abstract S2 — Abstract in Norwegian. (PDF) [file pone.0054356.s006.pdf]

Abstract S2: Abstract in Norwegian.

Høyt nivå av genomiske forandringer er assosiert med bedre overlevelse for pasienter med serøs ovariekreft

Genomisk ustabilitet og kopitallsendringer i kreftsvulster er vanligvis assosiert med dårlig prognose, men nyere studier tyder på at ekstreme nivåer av genomiske forandringer kan føre til bedre overlevelse for noen pasienter med enkelte tumortyper. Vi har undersøkt forekomst av genomisk ustabilitet i pasienter med serøs ovariekreft (SOK) i to uavhengige datasett fra Norge (n=74) og Australia (n=70). Den genomisk ustabiliteten ble kvantifisert ved å anvende Total Aberrationion Index (TAI), som maler hyppighet og genomisk størrelse av kopitallsforandringer i tumorer. I den norske kohorten hadde pasienter med TAI over medianen signifikant forlenget totaloverlevelse ($p<0.001$) og progresjonsfri overlevelse ($p<0.05$) sammenlignet med pasienter med TAI under medianen. I den australske kohorten hadde pasienter med TAI over median forlenget totaloverlevelse ($p<0.05$) og den progresjonsfrie overlevelsen var moderat forlenget, men ikke signifikant. Resultatene ble bekreftet i univariate og multivariate Cox regresjonsanalyser ved å bruke TAI som en kontinuerlig variabel. Våre resultater bekrefter at høyt nivå av genomisk instabilitet er av fordel for overlevelse for pasienter med SOK, kanskje på grunn av at ekstremt forstyrret genom-integritet, med økt sensibilitet for kjemoterapeutiske stoffer.
